# Supplementary material for: IRES-TrAPPr reveals novel insights into viral and cellular mRNA translation
Source: bioRxiv. 2026 May 9:2026.05.06.723280. Preprint. [Version 1] doi: 10.64898/2026.05.06.723280 (PMC13174604; doi:10.64898/2026.05.06.723280)
Supplement: Supplement 10 [file NIHPP2026.05.06.723280v1-supplement-10.pdf]

bioRxiv preprint doi: https://doi.org/10.64898/2026.05.06.723280; this version posted May 9, 2026. The copyright holder for this preprint (which was not certified by peer review) is the author/funder, who has granted bioRxiv a license to display the preprint in perpetuity. It is made available under aCC-BY 4.0 International license.

FIGURE S1:

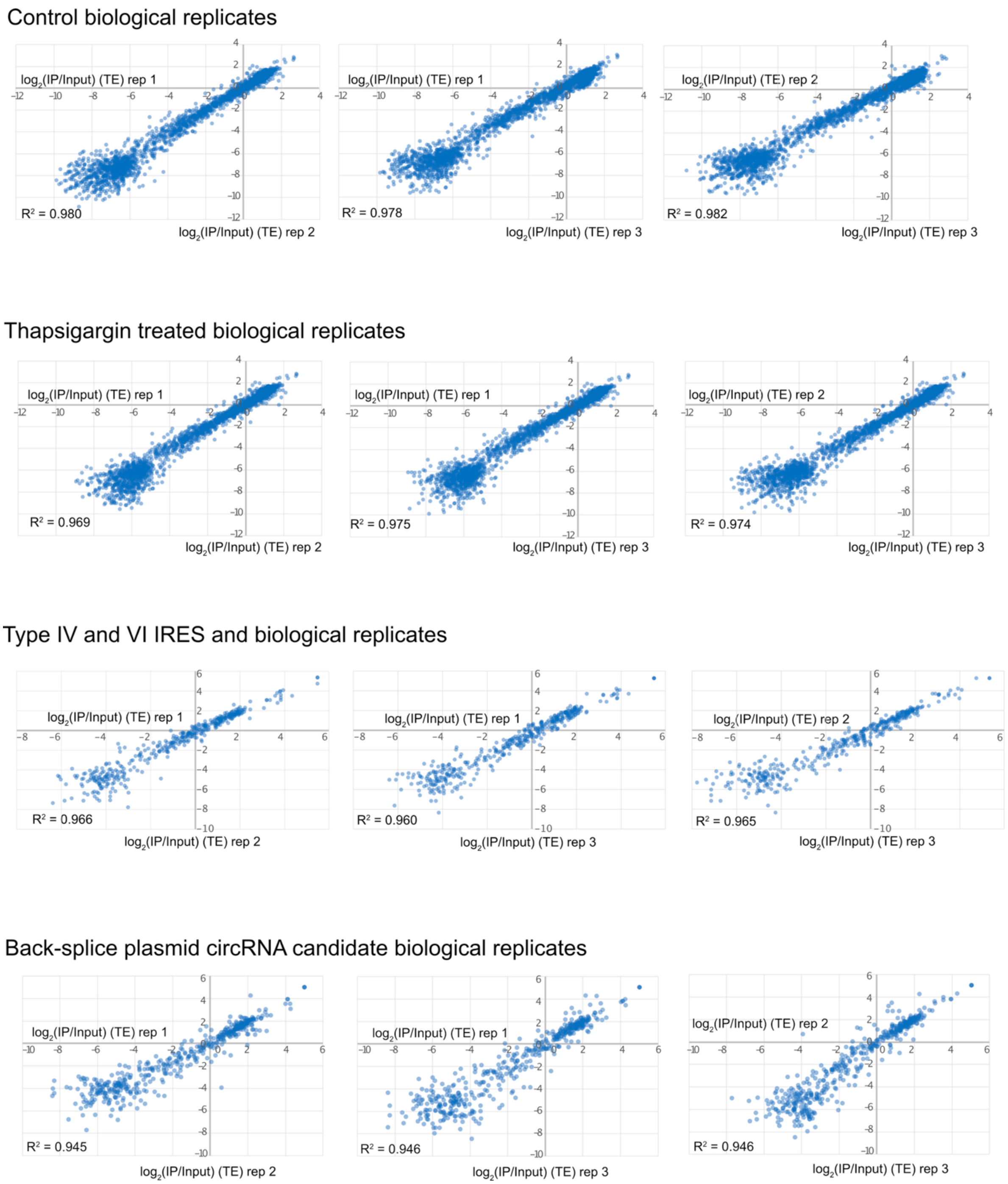

Figure S1. Comparison of biological IRES-TrAPPr replicates. Scatter plots show combined TE estimates for CDI and IRES activity for the experiments described in this manuscript. In all cases, there was strong correlation between replicates (R^2 >= 0.95).

bioRxiv preprint doi: https://doi.org/10.64898/2026.05.06.723280; this version posted May 9, 2026. The copyright holder for this preprint (which was not certified by peer review) is the author/funder, who has granted bioRxiv a license to display the preprint in perpetuity. It is made available under aCC-BY 4.0 International license.

FIGURE S2:

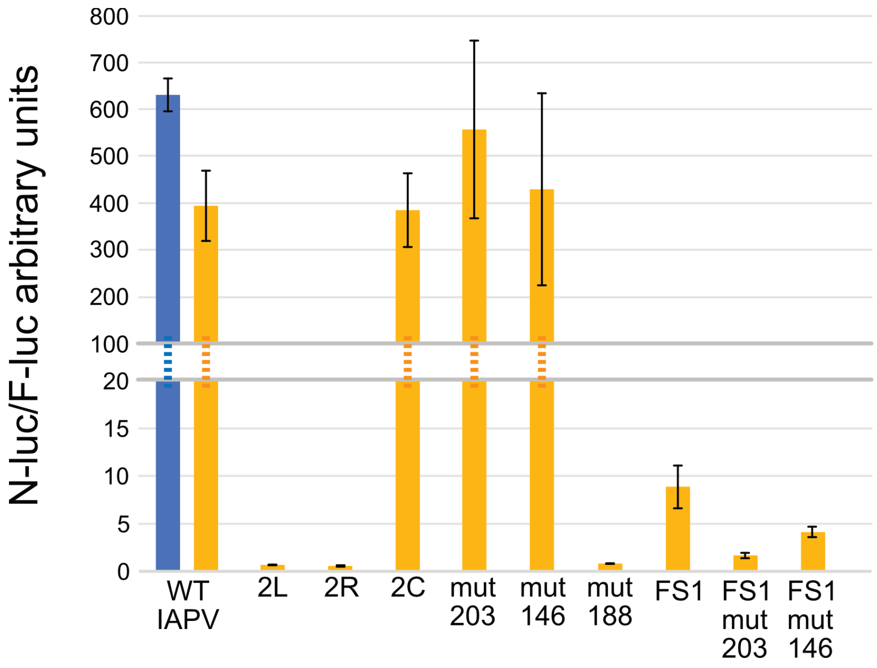

Figure S2. **Luciferase reporter validation of IAPV variants**. G-cap and A-cap (IRES) n-Luc reporter RNAs were transfected into HEK293T cells along with a common F-luc control (see methods). Mutations that disrupted basepairing in the second pairing region (2L and 2R) nearly eliminated IRES activity, while their combination (2C) restores basepairing and IRES activity. Mutations at positions 203 and 146 increased IRES activity in IRES-TrAPPr and individual reporters, however this was not statistically significant in individual assays. Mutation 188 decreased IRES activity in both IRES-TrAPPr and validation reporters. The FS1 mutation represents IRES activity in the +1 reading frame (n-Luc cloned in +1). FS1 mut 203 and mut 146 are +1 reading frame reporters with mutations at 203 and 146, both of which decrease +1 translation.

bioRxiv preprint doi: https://doi.org/10.64898/2026.05.06.723280; this version posted May 9, 2026. The copyright holder for this preprint (which was not certified by peer review) is the author/funder, who has granted bioRxiv a license to display the preprint in perpetuity. It is made available under aCC-BY 4.0 International license.

FIGURE S3.

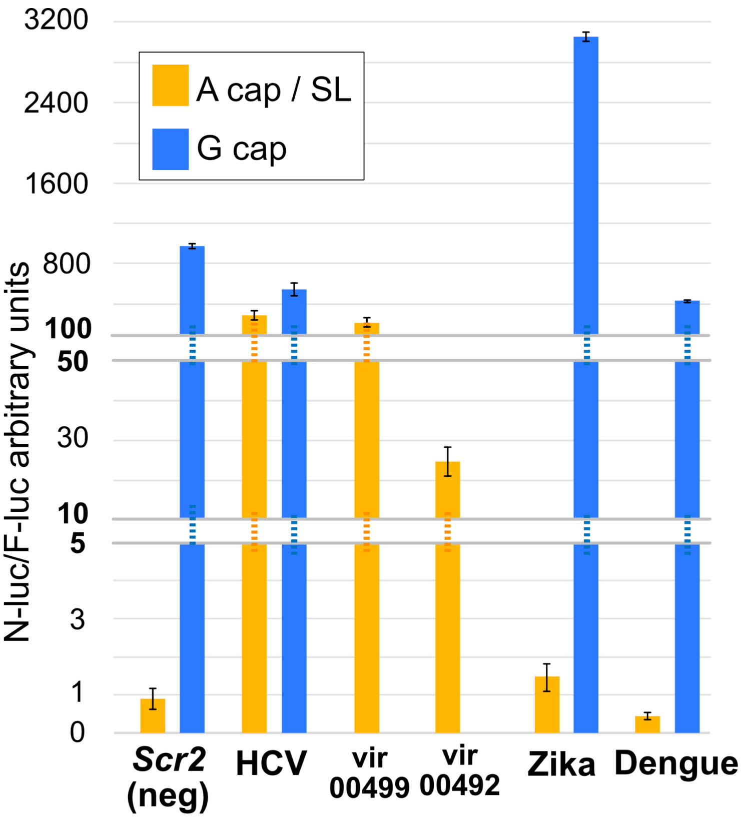

Figure S3. **Luciferase reporter validation of viral IRES candidates**. G-cap and A-cap (IRES) n-Luc reporter RNAs were transfected into HEK293T cells along with a common F-luc control (see methods). The two positive IRESbase candidates had strong IRES activity, while the 5' UTRs of Zika and Dengue virus had negligible activity.

bioRxiv preprint doi: https://doi.org/10.64898/2026.05.06.723280; this version posted May 9, 2026. The copyright holder for this preprint (which was not certified by peer review) is the author/funder, who has granted bioRxiv a license to display the preprint in perpetuity. It is made available under aCC-BY 4.0 International license.

FIGURE S4:

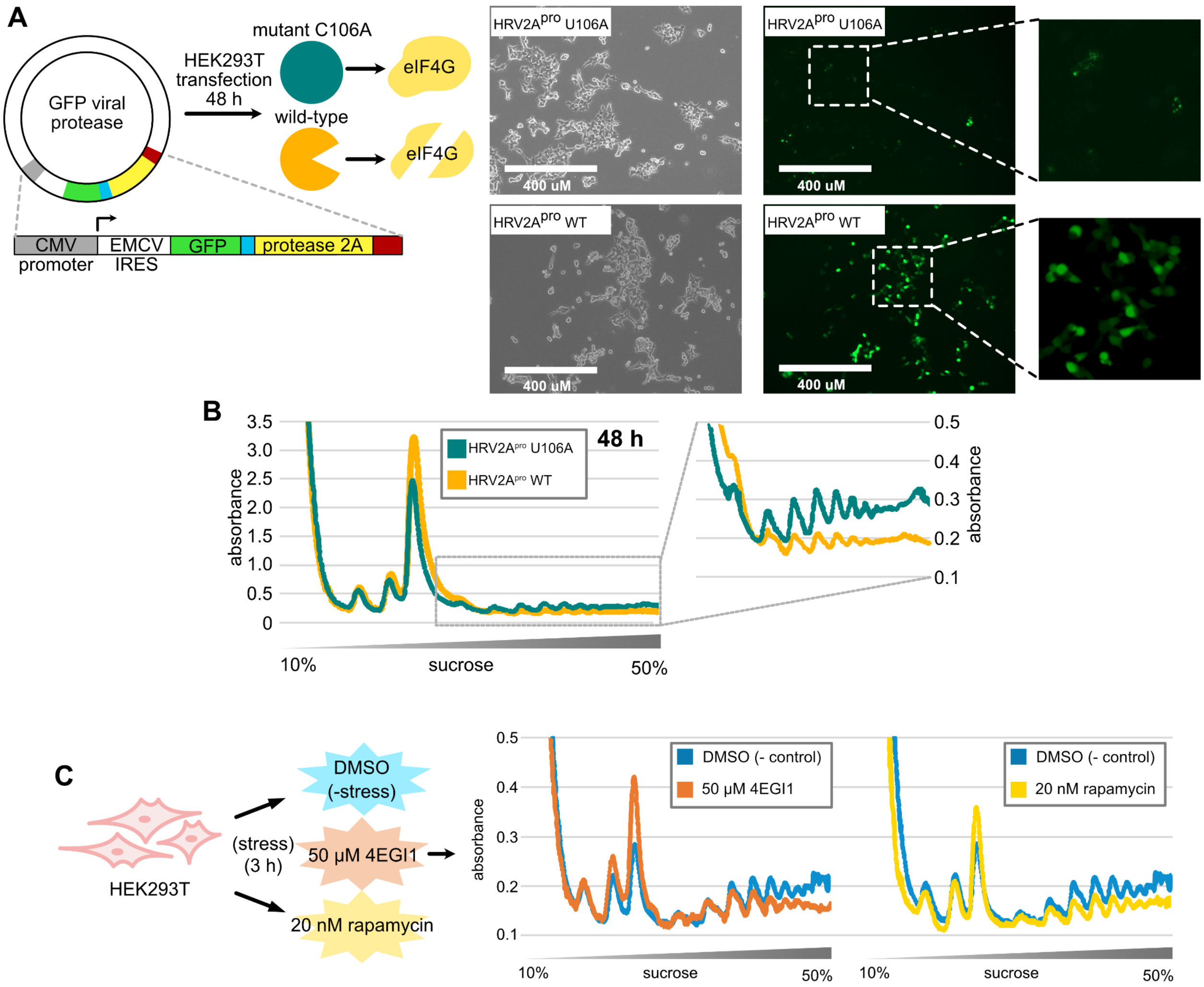

Figure S4. Expression of 2A protease and treatment with translational inhibitors induces translational stress. A. Plasmids encoding wildtype and nonfunctional U106A (Cys106Ala) Human Rhinovirus protease 2A fused to GFP were transfected into HEK293T cells. The blue segment in the diagram between GFP and the 2A protease is a 28 amino acid peptide recognized by the 2A protease for auto excision from GFP. Microscopy images of transfected cells show stable expression of GFP fused to wildtype, but not 2A mutant protease. **B.** Polysome gradient fractionation shows expression of 2A protease reduces polysomes and accumulates monosomes. **C.** Cells were separately treated with translation inhibitors 4EGI1 and rapamycin (left), which also reduced polysome accumulation and increased monosome abundance (right).
